# Camptodactyly Caused by an Anomalous Origin of the Flexor Digitorum Superficialis Tendon: A Case Report and Review of the Literature

**DOI:** 10.1055/s-0039-1697631

**Published:** 2019-11-13

**Authors:** Shkelzen B. Duci

**Affiliations:** 1 Clinic of Plastic and Reconstructive Surgery, University Clinical Center of Kosovo, Prishtina, Kosovo

**Keywords:** contracture, flexion, result, treatment

## Abstract

Camptodactyly is a flexion contracture of the proximal interphalangeal joints and is known as an isolated malformation that affects 1 in 300 in the population and can be inherited as an autosomal dominant trait with variable expression.

A 17-year-old female was referred to the Clinic of Plastic Surgery, University Clinical Center of Kosovo, Prishtina, for the first time with camptodactyly of the little finger in the right hand. She was presented with a progressive flexion contracture of the proximal interphalangeal joint greater than 110 degrees of her right little finger.

According to our observations from outpatient consultations, we concluded that the case of camptodactyly in the little finger in the flexible form (>110 degrees), which underwent surgical treatment, presented excellent result. Therefore, we think that the surgical technique used in our case report will contribute to treating this complicated deformity.

Camptodactyly, the flexion contracture of the proximal interphalangeal joint (PIP) is an isolated malformation that affects 1 in 300 in the general population and can be inherited as an autosomal dominant trait with variable expressions. 1 Most often, it occurs as a deformity of the little finger and may be bilateral. 2 3

In 1994, Benson et al 3 classified camptodactyly into the following three types:

- Type I: this is the commonest form of camptodactyly that becomes evident during childhood. It generally affects the little finger alone.
- Type II: camptodactyly during adolescence, which occurs predominantly in females, develops between 7 and 11 years of age, subtly initiating and gradually and progressively evolving. This type of camptodactyly generally does not spontaneously improve and may evolve to a severe flexion deformity.
- Type III: congenital camptodactyly, which usually affects several fingers, is associated with a variety of syndromes and other malformations. 4 5 6 7

## Case Report

**Fig. 1:**
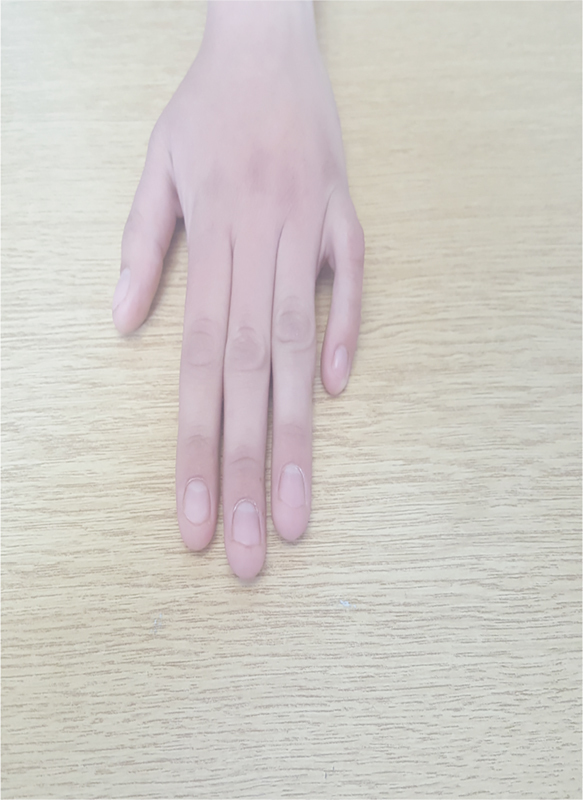
Camptodactyly (dorsal view).

**Fig. 2:**
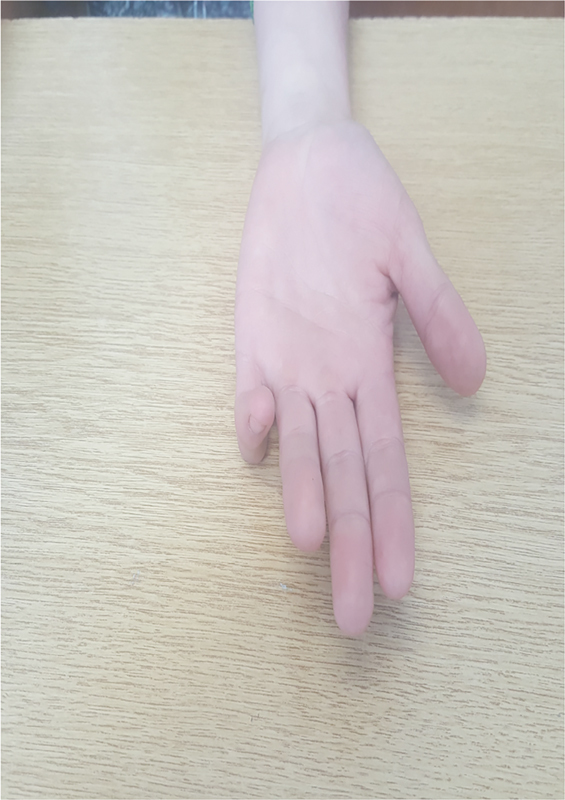
Camptodactyly with contracture of the proximal interphalangeal joint more than 110 degrees (palmar view).

A 17-year-old woman was referred to the Clinic of Plastic Surgery, University Clinical Center of Kosovo, Prishtina, for the first time with camptodactyly of the little finger in the right hand. She presented with a progressive flexion contracture of the PIP joint > 110 degrees of her right little finger ( Figs. 1 and 2 ). Her parents, who were unaffected by this disorder, had not observed this deformity of her finger during infancy.

**Fig. 3:**
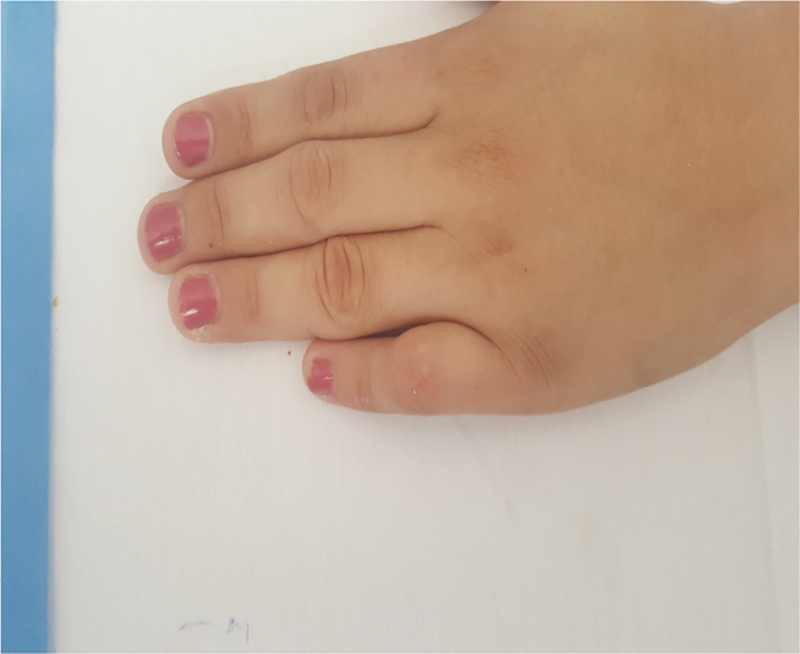
Camptodactyly of the right little finger 3 months after surgery (dorsal view).

In this case, we intraoperatively observed that the superficial tendon of the right little finger was hypoplastic and attached to the palmar aponeurosis. As per our understanding, the superficial flexor tendon of the little finger was the cause of the camptodactyly. After excision of the aberrant flexor tendon, the patient had a normal range of movement of the PIP joint; this supports our conclusion. Furthermore, capsulotomy in the PIP joint was performed to improve flexion contracture of the PIP joint, and the skin defect created in the palmar surface of the PIP joint after extension of the finger was simultaneously concealed by full-thickness skin graft that was obtained from the front of the right forearm. To prevent the recurrence of the deformity, we applied dynamic extension splint onto the operated finger. We followed the case for over a year after the intervention, and it presented with excellent results ( Figs. 3 4 5 6 ).

**Fig. 4:**
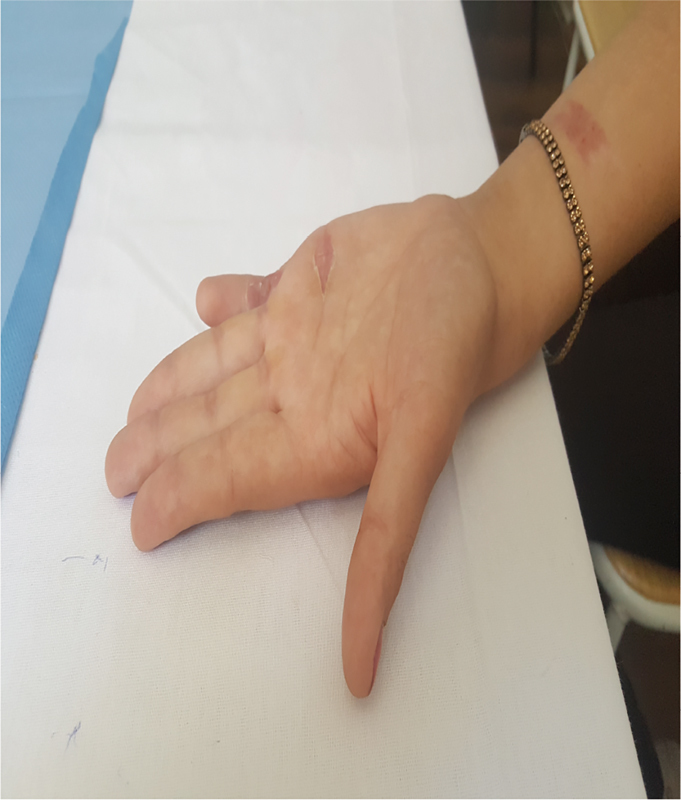
Camptodactyly of the right little finger 3 months after surgery (palmar view).

**Fig. 5:**
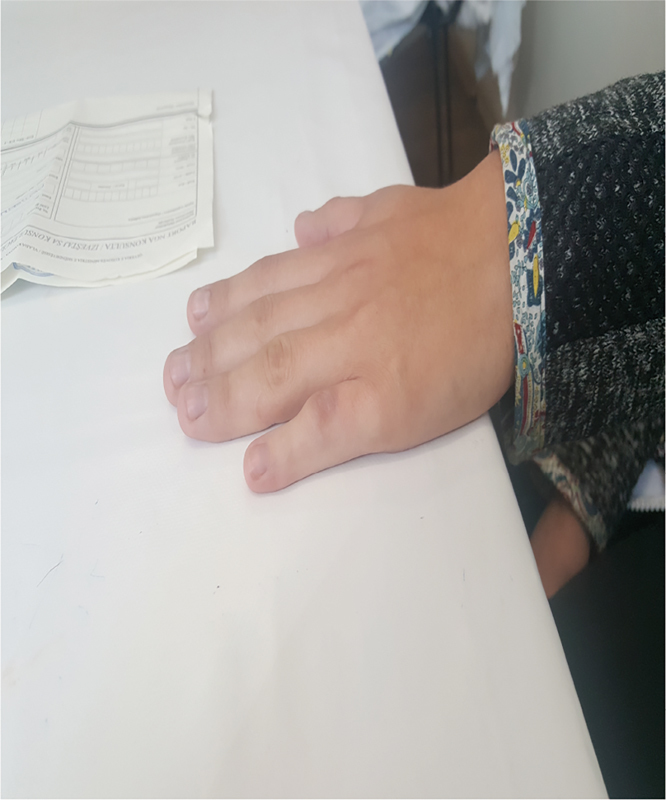
Camptodactyly of the right little finger 8 months after surgery (dorsal view).

**Fig. 6:**
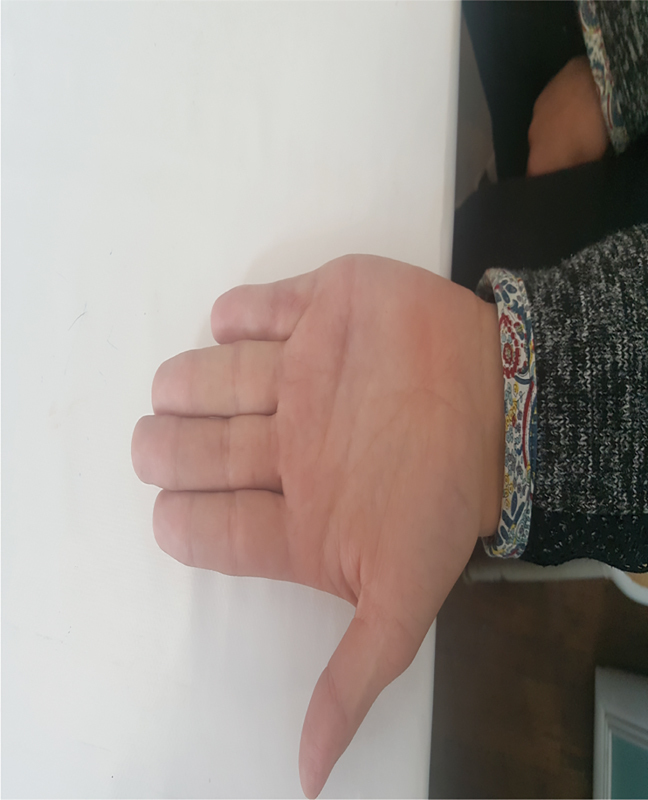
Camptodactyly of the right little finger 8 months after surgery (palmar view).

## Discussion

There are two types of camptodactyly: infantile type, in which a flexion deformity of the fingers observed during infancy and which equally involves both genders, and adolescent type, in which the deformity is not apparent during infancy but develops rapidly during adolescence and mainly involves young women. 8 The etiology of camptodactyly remains unknown; however, there have been several studies describing the conditions that contribute to this pathological deformation of the articular surface or the joint itself, of the volar plate or musculotendinous units, including the lumbrical muscles, flexor digitorum superficialis tendons, or the extrinsic and intrinsic mechanisms of the tendons. 9 10 11 12 13 14 15

Similar to our study, Hoogbergen et al 16 reported the case of a 20-year-old man with a progressive flexion contracture of the PIP joint of the right ring finger that had an anomalous origin of the flexor digitorum superficialis tendon. He underwent an excision of the aberrant flexor tendon, after which he had a normal range of movement of the PIP joint.

In another study conducted by Ogino and Kato, 17 the flexor digitorum superficialis tendon was found to be hypoplastic in five of six cases with camptodactyly, and there was no continuity of the normal tendon between the muscle belly and bony insertion. The authors suggested that the tenodesis effect of the abnormal tendon of the flexor digitorum superficialis is considered to play an important role in the incidence of the camptodactyly.

Conversely, McFarlane et al, 18 who noted abnormal insertion of the lumbrical muscle in 21 consecutive operations, supports the view of Millesi 19 that camptodactyly is caused by an imbalance between the flexor and extensor forces acting upon the PIP joint.

## Conclusion

The etiology of camptodactyly remain is unknown. However, various surgical techniques have been proposed for the treatment of camptodactyly. From outpatient consultations, we identified a dysfunctional superficial flexor tendon of the little finger in the flexible form (110 degrees) to be the cause of camptodactyly. The patient underwent surgical treatment and presented with a favorable outcome. Therefore, we believe that the surgical technique we used in this case will contribute, to an extent, to treating this complicated deformity.
